# Supplementary material for: Fatal intentional drowning in Australia: A systematic literature review of rates and risk factors
Source: PLoS One. 2020 May 22;15(5):e0231861. doi: 10.1371/journal.pone.0231861 (PMC7244177; doi:10.1371/journal.pone.0231861)
Supplement: Attachment — Submitted filename: Response to Reviewers.docx [file pone.0231861.s003.docx]

12-11-2019

Rohina Joshi
Academic Editor
PLOS ONE

Dear Rohina,

Re: PONE-D-19-21766 entitled " Fatal intentional drowning in Australia: A systematic literature review of rates and risk factors"

Firstly, we thank reviewers for their work in reviewing our systematic literature review entitled “Fatal intentional drowning in Australia: A systematic literature review of rates and risk factors” and providing their feedback. We have noted our responses to the suggested revisions in the table below as well as in the marked up version of the manuscript in track changes. As requested, we have also uploaded a clean version of the revised manuscript.

We hope that you find our responses satisfactory. It would be an honour to be published in the PLOS ONE.

Kind regards,

Richard Franklin (corresponding author)

James Cook University

richard.franklin@jcu.edu.au

| Reviewer Number | Comment | Author response |
| --- | --- | --- |
| Journal requirements | | |
|  | Please ensure that your manuscript meets PLOS ONE's style requirements, including those for file naming. | Thank you, we have ensured in the uploading of the new files, that they meet the style requirements including file naming. |
|  | 2. Please include captions for your Supporting Information files at the end of your manuscript, and update any in-text citations to match accordingly. Please see our Supporting Information guidelines for more information: <http://journals.plos.org/plosone/s/supporting-information>. | We have included captions for the supporting information files on page 30 and revised the in-text citations to ensure they match. Please see the revised manuscript. |
| Reviewer 1 | | |
|  | 1. Page 4 line 54 – I believe the most up to date drowning figure from WHO is 360,000 deaths per year. Please check latest WHO data on drowning. | Thank you for this comment. We have revised the current estimate to over 320,000 in accordance with the most recent data on the WHO website. Please see the revised manuscript. |
|  | 2. Page 4 line 56 – Please say upfront what the discrepancy is in this sentence – that WHO may be an underestimate | Thank you for this comment. We have amended this sentence for clarity as suggested, and it now reads as follows:  “However, the drowning statistics reported by the WHO may underestimate the actual magnitude of drowning as has been suggested in several previous studies.(5-8)” |
|  | 3. Page 4 lines 63 onwards – It is not clear from this introduction if you will be focussing on suicidal only, or also assaults? What do we know about assaults v suicide in terms of proportion of intentional drownings? Would be good to include to make it clear what the scope is, and that it’s important to consider both types of intentional drownings. | This review focused on intentional drownings, that is both suicide and assault, though no publications explored assault. We have clarified this in the revised aim as follows:  “Through a systematic review of the literature, this study aimed to describe the epidemiology, the mortality rates and risk factors, of intentional drowning deaths in Australia (i.e. both suicide and assault). This study answers these following questions:” |
|  | 4. Page 5 line 77 – Please capitalise report titles | Thank you for this suggestion. The report title has been capitalised. Please see the revised manuscript. |
|  | 5. Page 5 line 100 – The question is ‘What are the risk factors’ rather than just identifying if that evidence for risk factors exists | Thank you for this suggestion. We have rewritten the research question as follows:  What are the risk factors for fatal intentional drowning in Australia? |
|  | 6. Page 5 line 99 - Also, the proportion being investigated is the proportion of drowning as a cause for suicide, rather than the proportion of total drowning that are intentional. Please make this clear. | Thank you for this suggestion. .We have added another research question which reads as follows:   - “What proportion of suicide mechanisms is drowning in Australia?” |
|  | 7. Page 6 line 107 – What do you mean exactly by the most exhaustive? | We mean that these search strings returned the highest number of results. We have revised this sentence as follows:  “The most exhaustive search strings (i.e. those that returned the highest number of results) were applied in the systematic search, in accordance to each database utilised (Table 1).” |
|  | 8. Table 2 – As per the aim in the Abstract, the objective is identify risk factors and mortality rates. In that case, was one of your inclusion criteria that the article needed to have calculated or assessed at least one of these in relation to intentional drowning? | Thank you for identifying this. We have added the following point to Table 2 in the exclusion criteria column:  Reported mortality rates and/or risk factors.  Please see the revised manuscript. |
|  | 9. Table 2 – If the article discussed both unintentional and intentional drowning, what was done? | Thank you for this query. Where an article discussed both, it was included, if the intentional drowning deaths could be extracted from the total. We have clarified this in the text above table 2 as follows:  “If a study examined both intentional and unintentional drowning cases, it was included if intentional drowning rates and risk factors could be differentiated from the unintentional cases.” |
|  | 10. Line 122 Page 7 – To be clear, how was the ‘substantiality’ assessment of the article done, what does this mean? Was this done with the McMaster Guideline as well? The sentence structure makes it appear substantiality and methodological quality are two different assessments you made. If not, please provide more information on what substantiality means and how it was assessed, otherwise please restructure the sentences. | Thank you for the comment. For the use of the word ‘substantiality’, we mean that, by using the McMaster Guideline, we reviewed whether the results of the study used the appropriate analysis method(s) and reported the clinical importance of the study, in relevance to our study objectives, which is also a part of the assessment of the methodological quality of each study.  Hence, we have amended this sentence for clarity, and it now reads as follows:  “The full-text version of the identified articles was then appraised to examine the methodological quality of the studies, using the McMaster appraisal guideline for quantitative and qualitative studies.” |
|  | 11. Page 12 – Please cite Donaldson with year in-text and without the first name | Thank you for this comment. Bi is the second author, not Donaldson’s first name. We neglected to include the third author so this now reads as follows:  Donaldson, Bi and Hiller, 2007 |
|  | 12. Page 23 – Perhaps another limitation may be the timelines of the studies – bulk of which are almost 10 years old or older. The need for up to date research may be highlighted. | Thank you for this suggestion. We have added the following sentence highlighting this point, to the end of the strengths and limitations section:  The current study also highlights the need for more up-to-date research on intentional drowning in Australia, with the bulk of the studies included in this review 10 years old or older. |
|  | 13. Would be useful to make a statement somewhere on the consistency of findings between the included studies – e.g. whether similar mortality rates / suicide proportion / risk factors have been found, and what this says about the quality and types of data available and need for further research. | Thank you for this comment. We acknowledged the consistency of finding between the included studies in the Strength and Limitation section, with these sentences:   - “This study also shown the consistency of the source of data, with 66.67% of the articles identified in this study using the national-scale data from the ABS, AIHW, and NCIS, displaying the potential of utilisation of the national-scale data, with some improvements on the data collection system as discussed the Recommendation for Future Research section, for further investigation on intentional drowning death in Australia.” - “Similar proportions of suicide by drowning also noted between the included studies with national-scale source of data (48, 49, 55), with drowning identified as the preferred method of suicide in 2% to 3% of all suicide deaths in Australia.” - “Furthermore, no studies identified in this current study investigated drowning deaths due to assault, highlighting the lack of peer-reviewed publications on homicidal drowning deaths in Australia and the need to further research on the topic in the future.” |
|  | Overall writing comments - Please read through the document and ensure that the sentences are shorter. For example lines 57-61 on page 4 is cumbersome to read. Some other sentences are like this. Better to split these into separate sentences. Another such confusing sentence is on Page 15 line 11 onwards. | Thank you for this comment. We have given the paper another thorough proof read to pick up any long sentences and have broken then up to shorten them and help with readability. Please see the revised manuscript. |
|  | - Discussion was very well thought out and analysed. It can be made more succinct by grouping together the studies you have discussed that have similar findings. | Thank you for this comment. We have given the Discussion another thorough read and grouped similar findings together and shorten the paragraphs to make the Discussion more succinct. Please see the revised discussion session. |
| Reviewer 2 | | |
|  | The manuscript is a systematic review of intentional drowning in Australia of rates and risk factors. The review includes 10 studies | No further action required. |
|  | Abstract The methods in the abstract say 2007 to 2018, but the results report trend on 1998- 2012 based on 8 studies. Am a bit confused- given the inclusion criteria findings should be limited to defined period. | Thank you for this comment. The review looks at studies with a published date from 2007 to 2018, regardless of the time period of the data reported in the study. The studies spanned 1907-2012 which we have added to the results in the abstract. The annual rate can only be inferred from the included studies between 1994 and 2001, which the abstract already says. |
|  | Given the limited of studies and the details of methods of included study- perhaps it is more appropriate to call it a narrative synthesis. Risk factors are fundamentally demographic descriptive distribution of intentional drowning deaths. | Thank you for this suggestion. However, the number of studies does not dictate the type of review that was undertaken. A systematic literature review was undertaken and as such we would prefer to retain that in title and manuscript. |
|  | Introduction Minor Line 67- uses both singular and plural, please revise | Thank you for this suggestion. We have edited the sentence as follows:  “Despite the limited number of publications, a high proportion of intentional drowning deaths were identified amongst all fatal drowning incidents reported in previous studies.(21-26)” |
|  | Line 74- uses “suicide- suicidal” twice. Using self- harm would read better. | Thank you for this suggestion. We have replaced the second use of suicide with self-harm as suggested and it now reads as follows:  …deaths due to self-harm registered in the region |
|  | Line 86- Except for Reference 9- the other reference are for suicide and psychiatric disorder in general, and not for drowning. The sentence is misleading in that it implies “suicide by drowning” is a unique case. | Thank you for this comment.  Reference 33-36 reported the relationship between suicide by all methods, included by drowning, and psychiatric disorders. |
|  | Line 96- Title states “fatal” so epidemiology would be for mortality- “particularly…” is perhaps not needed. | Thank you for this suggestion. We have removed the word ‘particularly’ here. Please see the revised manuscript. |
|  | Second point in aims, line 100- “Is there any evidence available on the fatal intentional drowning risk factors in Australia?” is more suitable for evidence gap maps rather than SR. In my understanding from the abstract the abstract reports on risk factors for intentional drowning. | Thank you for this comment. In line with your comment and that of reviewer 1, we have reworded this point to read as follows:   - “What are the risk factors for fatal intentional drowning in Australia?” |
|  | Results Line 148- reference 54- based on the study objective of Ref 54 and the inclusion criteria mentioned in Table 2; this Is not a primary study in my opinion should not be included in the SR. Can the authors please explain a bit- on the rationale for including this study. | Thank you for this comment.  Reference 54 is included within this study as it fits the inclusion criteria:   - Original research article (methods: data source were all media reports that mentioned completed suicides from 515 sources encompassing all Australian metropolitan daily newspapers, a sample of suburban news media and all national radio and television networks between March 1, 2000, and February 28, 2001). - Investigating intentional drowning deaths, in this case suicide drowning, in proportion of all drowning cases by all methods reported in the news. - A peer-reviewed journal article published in 2007 (the inclusion criteria: published between 2007-2018) - Specifically took place in Australia - Observational studies - Full-text available - Published in English   Hence, we would prefer to retain this study and as such have made no alterations to the text in response to this suggestion. |
|  | Page 12 does not have line numbers. The first paragraph again reports on findings beyond the study period It is hard to take away anything from the analysis section | Thank you for this comment. The landscape tables have interrupted the line numbering, apologies for that. As clarified in the abstract, the inclusion criteria was published date, not study period. As such, these findings are within the scope of reference for this review.  The analysis was performed on these aspects of the studies included: The year of study and publication, data source, study design, scale of study, manner of intentional drowning deaths investigated, and mortality rates and risk factors of intentional drowning in Australia. |
|  | Table 5 is not particularly helpful- and repetitive on text. | Thank you for this comment. We feel that this table does add value in that it allows for comparison of trends over time between the included studies. We would prefer to retain this table and as such have made no alterations to the text in response to this suggestion. |
|  | Page 15 line 7 & 8- is the risk being compared for drowning suicide or only suicide? | Thank you for this comment.  The risk of older adults for suicidal drowning. |
|  | Discussion - Table 6- does the journal accept table in discussion. Is repetitive of text above the table. | Thank you for this comment. We would like to retain the table in its current position. However if the journal’s formatting is not to accept tables in the discussion, we can remove it. |
|  | Discussion is fair bit repetition of the results. The authors refer to need for different types of intervention for intentional vs unintentional drowning deaths in the intro but do not come back / reflect on the same in the discussion. Presumably there is no study or data for indigenous populations- perhaps discuss why? | Thank you for the valuable input regarding the Discussion section.  Within the Discussion section, under the sub-section for Recommendation for Future Research, we acknowledged the gap identified within the studies included in this review, which is the under-exploration of risk factors of intentional drowning deaths.  “By recommending the investigation of the association of intentional drowning and several factors ((1) socio-demographic characteristics, including age, gender, socioeconomic status, unemployment, rurality, and indigenous identification; 2) substance dependence; 3) psycho-pathology, including depressive disorders, psychotic disorders, other neurotic disorders, and previous intentional self-harm attempts ; and 4) social dysfunction, including history of childhood abuse, social isolation, and homelessness) in future research, a better understanding on the association of fatal intentional drowning and these risk factors can contribute to the development of public policy and the enhancement of community action in creating supportive physical and social environment for mental health and intentional drowning prevention promotion in Australia. This conjunction of the enhancement of mental health promotion and deliberate self-harm prevention most likely differs from the development of unintentional drowning prevention strategies.”  Regarding the indigenous identification, there are 3 studies included in this review (48, 55, 56) that explore indigenous status within their studies (suicide victims of all methods), however none of these studies explored indigenous suicide by drowning. Hence, we did not discuss suicide drowning in indigenous populations in Australia and put the recommendation to investigate the relationship of intentional drowning and indigenous status, within the Discussion section, under the sub-section for Recommendation for Future Research. |
