# Supplementary material for: Fatal intentional drowning in Australia: A systematic literature review of rates and risk factors
Source: PLoS One. 2020 May 22;15(5):e0231861. doi: 10.1371/journal.pone.0231861 (PMC7244177; doi:10.1371/journal.pone.0231861)
Supplement: Attachment — Submitted filename: Response to Reviewers.docx [file pone.0231861.s004.docx]

April 2, 2020

Laura Schwab-Reese

Academic Editor

PLOS ONE

Dear Laura,

Regarding PONE-D-19-21766R1 ‘Fatal intentional drowning in Australia: A systematic literature review of rates and risk factors’

Firstly, we thank the reviewer for the work in reviewing our systematic literature review entitled “Fatal intentional drowning in Australia: A systematic literature review of rates and risk factors” and providing their feedback. We have noted our responses to the suggested revisions in the table below as well as in the marked-up version of the manuscript in track changes. As requested, we have also uploaded a clean version of the revised manuscript.

We hope that you find our responses satisfactory. It would be an honour to be published in the PLOS ONE.

Kind regards,

Richard Franklin (corresponding author)

James Cook University

[richard.franklin@jcu.edu.au](mailto:richard.franklin@jcu.edu.au)

| Reviewer Number | Comment | Author response |
| --- | --- | --- |
| Reviewer 1 | | |
|  | 1. Page 2 line 28 - 1907 does not come in your range of 2007 to 2012 please clarify that this is data and not the paper itself | Thank you for this comment. Yes, it is indeed the study periods of the articles reviewed in this study, instead of the timeframe of this current study. Therefore, we have amended the sentence for clarity, and it now reads as follows:  “Ten papers reporting the mortality rates and risk factors of intentional drowning deaths in Australia published between 2007 and 2018, with study periods of the included articles spanning from 1907 to 2012, were reviewed.”  Please see the revised manuscript. |
|  | 2. Page 2 line 35 - Please include the finding on what proportion of suicide deaths are from drowning – this is an important finding and of interest given the focus on suicide | Thank you for this comment. We have added an additional statement regarding the proportion of suicide deaths from drowning, in comparison to all intentional self-harm fatalities, as suggested:  “The national-scale proportion of suicide drowning in Australia ranging from 2% to 3% of all intentional self-harm deaths was also identified.”  Please see the revised manuscript. |
|  | 3. Page 4 line 55 - LMIC context is not important to mention as Australia is not LMIC | Thank you for identifying this. We have omitted the statistics of drowning in LMICs, and it now read as follows:  “According to the World Health Organization (WHO) Global Report on Drowning, 2014 (3) annually, the lives of 372,000 people are lost due to drowning worldwide. A recent study on the global, regional and national burden of unintentional drowning mortality from the Global Burden of Disease 2017 by Franklin et al, 2020 (1), reported a global estimate of 295,210 fatalities was attributed to drowning.”  Please see the revised manuscript. |
|  | 4. Page 4 line 58-62 - Can be summarised more succinctly and focus more on the Australian studies. Otherwise, justify why you are talking about these countries in detail in particular and why a comparison is appropriate and useful. | Thank you for this suggestion. The paragraph has been revised to focus more on the Australian studies and the studies performed in other countries have been omitted from the paragraph, and now it reads as follows:  “However, the drowning statistics reported may underestimate the actual magnitude of all drowning deaths as has been suggested in several previous studies, particularly regarding unintentional drowning fatalities.(5-8) Peden et al, 2017 (6) identified a 40% under-report between WHO methodology and unintentional fatal drowning in Australia. A similar study also estimated a 35% higher unintentional drowning mortality rate in Australia and the United States (US) when compared to WHO estimates, with other studies estimating a gap of 50%.(7) The recent study on the burden of unintentional drowning mortality from the Global Burden of Disease 2017, including in Australia, also did not include intentional drowning, which also underlines the need for further study on intentional drowning deaths.(1)”  Please see the revised manuscript. |
|  | 5. Page 4 line 73 - Would not say conflicting – there are varying proportions | Thank you for this suggestion. We have rewritten the statement and now it reads as follows:  “The evidence on the proportion of intentional drowning of all suicidal deaths, however, shows varying proportions across the world.”  Please see the revised manuscript. |
|  | 6. Page 4 line 71-72 - Am not sure in what population you are talking about for this finding | Thank you for this comment. We have revised the statement as follows:  “In contrast to the disproportionately higher risk for unintentional drowning amongst male populations worldwide, more than half of suicide drowning cases in Sweden were performed by females (55% versus 21%, p<0.001). (2, 27)”  Please see the revised manuscript. |
|  | 7. Page 5 line 84-85 - Why exactly is it important to identify this? Please elaborate a little. | Thank you for this comment. We have elaborated the importance on identifying the trend of intentional drowning deaths in Australia:  “It is important to determine whether a similar trend is also observed for intentional suicide by drowning deaths in Australia. This will provide a better understanding on whether suicide drowning deaths have been contributing to the increasing trend of suicide mortality rate in Australia, underlining the urgency of advocating for the enhancement of appropriate preventive measures for intentional drowning fatalities. This may differ from unintentional drowning prevention efforts. (32)”  Please see the revised manuscript. |
|  | 8. Page 7 line 124 - Please specify that exclusion criteria is ‘Only unintentional drowning’ | Thank you for the comment. We have revised the exclusion criteria, as suggested. Please see the revised manuscript. |
|  | 9. Page 8 line 142 - The parentheses are a little unclear, I had to read it a few times to understand what you meant. Please restructure the sentence. | Thank you for this comment. We have revised the sentence to:  “Of ten articles included in this review, only one article (50) reported mortality rates of suicide drowning in Australia, while the majority of articles reviewed (48-50, 52-57) investigating suicidal drowning deaths in proportion to suicidal deaths of all causes in Australia.”  Please see the revised manuscript. |
|  | 10. Page 12 - Can you please provide detail on how you used the results from papers looking at just one state, such as NSW or Qld. How were these rates converted and included in your national rate calculation? | Thank you for the comment. The nation-wide annual rates of intentional drowning were inferred from studies (48, 53, 54, 55) that report nation-wide suicide drowning deaths and sourced their data from national scale sources (e.g. ABS, NCIS, AIHW, news reports). These rates were inferred by extracting the number of intentional drowning deaths from the papers in proportion to the population of the said year based on Australian population data provided by the ABS.  Meanwhile, **the studies which only reported state-wide rates of intentional drowning deaths, including for QLD (52, 56), Victoria (51) and NSW (49) were not included in the nation-wide mortality rate calculations.** Instead, these state-wide rates were inferred by extracting **the number of intentional drowning deaths in the state (QLD, Victoria, or NSW) from the paper in proportion to the population of the corresponding state for the said year (based on ABS data).**  We have restructured the paragraphs to provide more clarity on how the nation-wide or state-wide mortality rates were calculated, and also added additional explanation on these calculations in the Methods section. Please see the revised manuscript and Table 5 for more detailed information. Please be informed that Table 5 included inferred annual national- and state-wide mortality rates of intentional drowning in Australia between 1994 and 2012, and we have modified the table to provide more clarity for the readers. |
|  | 11. Page 16 line 35 - You have spent a lot of space discussing other high-income countries – what is the conclusion you want the reader to come to? Please tell us the ‘so what’. | Thank you for this comment. We have added the following sentences highlighting several conclusions that we hope the readers will be able to come to:  “The dearth of epidemiological studies of intentional drowning deaths in high-income countries, including in Australia, underlines the gap between the more thoroughly understood epidemiology and advanced efforts of preventing unintentional drowning deaths, in comparison to the relatively under-exposed and under-represented intentional drowning deaths. This also highlights the possibility of further under-exploration of the intentional drowning deaths in the resource-limited setting of developing nations, where injury surveillance system, including for self-harm related events, might not be as well-developed as those available in high-income countries. (70, 71)”  Please see the revised manuscript. |
|  | 12. Page 17 line 51 - Are there any theories as to why this proportion is lower in Australia? This seems a little counter intuitive given Australian’s better access to water bodies. | Thank you for this comment. We haven’t found any study that explained this discrepancy however we have added that the lower proportion of intentional drowning in Australia, in comparison to other developed nations, and we noted in the manuscript that it is an area needing more research and now it reads as follows:  “There is a dearth of publications explaining the discrepancy between the lower proportion of suicide drowning among all suicide deaths in Australia in comparison to other developed nations. It is noted that there is a need for further research addressing the preference for suicide methods by countries, to inform the development of intervention strategies appropriate for each country, including Australia.”  Please see the revised manuscript. |
|  | 13. Page 17 line 58 - Whilst the discussion on risk factors is interesting, it could perhaps be shortened. As these are only based on one study for each of the risk factors, there are no real additional learnings from the systematic review on this paper’s findings – you are reiterating what is already in the original paper. Where there is additional knowledge to be gained from bringing together more than one paper, this should be elaborated upon. | Thank you for this comment. We have given the Discussion another thorough read and shortened it to make the Discussion more succinct. Please see the revised discussion session. |
|  | 14. Page 22 line 175 - If there are 10 papers, 67% can’t be the percentage. | Thank you for identifying this discrepancy. It is indeed 8 out of 10 papers (80%) identified in this study to be sourced from the national-scale data from the ABS, AIHW, and NCIS. We have made the appropriate revision, please see the revised manuscript. |
|  | 15. Page 22 line 183-185 - This sentence is unclear – not sure what you mean | Thank you for this comment. We realised that the sentence is cumbersome, revised it, and now it reads as follows: “However, several major limitations were identified in this review.”  Please see the revised manuscript. |
|  | Overall writing comments - English needs polishing – there are several sentences that don’t make much sense on first reading. Would suggest reading it out loud before re-submitting. | Thank you for this comment. We have given the paper another thorough proofread to pick up any long sentences and have broken them up to shorten them and help with readability. Please see the revised manuscript. |
|  | - Figures are very unclear to read – not sure if this will be the case in finally online version | Thank you for this comment. We will provide separate Figure files with better resolution within the submission of the revised manuscript. Please zoom into the figures to see them more clearly. |
|  | - There should be some acknowledgement that overall this type of suicide is not a major issue. I would suggest you comment on how specific interventions to target this exact type of suicide may not be a priority, but if common risk factors/ precursors appear with other suicide types, then intentional drownings can also be prevented along with other types of suicides. | Thank you for this comment. We have included this statement, as suggested, under the Discussion and Conclusion section:  “It is relevant to acknowledge that this current review identified suicide by drowning as the preferred method of suicide in only 2% to 3% of all intentional self-harm deaths in Australia. This highlights the importance of early recognition of mental disorders and previous suicide attempts, as well as the enhancement of mental health promotion and deliberate self-harm prevention, as a part of intentional self-harm and suicide prevention strategies, including for suicide by drowning.”  Please see the revised discussion session. |
